# Disseminated Cryptococcosis Revealing an HIV Infection: A Case Report

**DOI:** 10.7759/cureus.37403

**Published:** 2023-04-10

**Authors:** Yassine Akrim, Hicham Ouasif, Hind Zrikem, Awatif El Hakkouni

**Affiliations:** 1 Biology Department, Parasitology and Mycology Laboratory, Mohammed VI University Hospital of Marrakech, Faculty of Medicine and Pharmacy of Marrakech, Cadi Ayyad University, Marrakech, MAR; 2 Biochemistry-Toxicology Laboratory, Avicenna Military Hospital, Marrakech, MAR; 3 Biology Department, Medical Analysis Laboratory, Mohammed VI University Hospital of Marrakech, Faculty of Medicine and Pharmacy of Marrakech, Cadi Ayyad University, Marrakech, MAR

**Keywords:** hiv, cryptococcal pneumonia, cryptococcal meningitis, cryptococcus neoformans, disseminated cryptococcosis

## Abstract

Cryptococcosis is a common fungal infection regarded as a disease of immunocompromised patients with high mortality. Cryptococcosis is usually observed in the central nervous system and lungs. However, other organs may be involved such as skin, soft tissue, and bones. Disseminated cryptococcosis is defined as fungemia or the involvement of two distinct sites. Here, we report the case of a 31-year-old female patient with disseminated cryptococcosis with neuro-meningeal and pulmonary involvement revealing a human immunodeficiency virus (HIV) infection. Chest computed tomography scan showed a right apical excavated lesion, pulmonary nodules, and mediastinal lymphadenopathy. Concerning biological tests, hemoculture, sputum, and cerebrospinal fluid (CSF) culture were positive for *Cryptococcus neoformans*. The latex agglutination test for cryptococcal polysaccharide antigen was positive in CSF and serum and HIV infection was confirmed by serological testing. The patient did not respond to initial antifungal therapy with amphotericin B and flucytosine. Despite the adaptation of antifungal treatment, the patient died of respiratory distress.

## Introduction

Cryptococcosis is an invasive and opportunistic fungal infection caused by *Cryptococcus neoformans* or *Cryptococcus gattii*, typically encountered in immunocompromised patients and among those with human immunodeficiency virus (HIV) infection [1]. *C. neoformans* has a cosmopolitan distribution and is usually isolated from bird droppings. On the contrary, *C. gattii* is frequently reported in tropical and subtropical areas and is associated with eucalyptus trees [2]. Meningoencephalitis is the most frequent manifestation of cryptococcal disease [3]. *C. neoformans* is life-threatening by dissemination into the central nervous system (CNS), *C. gattii* commonly causes a severe pulmonary infection without dissemination [4]. Disseminated cryptococcosis is defined as the simultaneous infection of two or more non-contiguous organs [5]. The diagnosis of cryptococcosis is usually made by detecting the capsulated yeast or the cryptococcal antigen test from biological fluids [6].

Here, we report a case of disseminated cryptococcosis with neuro-meningeal and pulmonary involvement revealing an HIV infection.

## Case presentation

A 31-year-old female patient presented to the emergency department with complaints of continuous and gradually worsening headaches over the past three months. The headache was holocranial and evolved in the context of fever and a deterioration of the general condition (asthenia, anorexia, weight loss). Two weeks later, the patient developed sudden and persistent hearing loss, vomiting, cough, and dyspnea. She also reported a history of pulmonary tuberculosis treated successfully six years back.

Physical examination revealed a febrile and conscious patient who was hemodynamically stable. The neurological examination revealed a nuchal rigidity and Brudzinski’s sign. During the pulmonary examination, she was hypoxic and required oxygen therapy to maintain saturation. Moreover, crackling lung sounds were noted on pulmonary auscultation. No abnormality was noted in the other systems.

Blood investigation showed a low lymphocyte count of 430 cells/mm^3^ and a high C-reactive protein (CRP) of 155 mg/L. The results of the other tests were normal (Tables 1, 2). The diagnosis of HIV infection was confirmed by serological testing.

**Table 1:** Complete blood count at presentation.

Test	Results	Reference range
Hemoglobin	13.8	12–14 g/dL
Mean corpuscular volume	85	80–100 fL
Packed cell volume	38	35–45%
White blood cell count	9.9	4–10.5/μL
Lymphocyte count	0.430	1.5–4/μL
Platelets	221	150–400/μL

**Table 2:** Comprehensive metabolic panel at presentation.

Test	Results	Reference range
C-reactive protein	155	<6 mg/L
Sodium	142	135–145 mmol/L
Potassium	3.9	3.5–5 mmol/L
Chloride	107	98–108 mmol/L
Bicarbonate	24	22–28 mmol/L
Blood urea nitrogen	12	5–20 mg/dL
Creatinine	0.7	0.6–1.2 mg/dL

**Figure 1:**
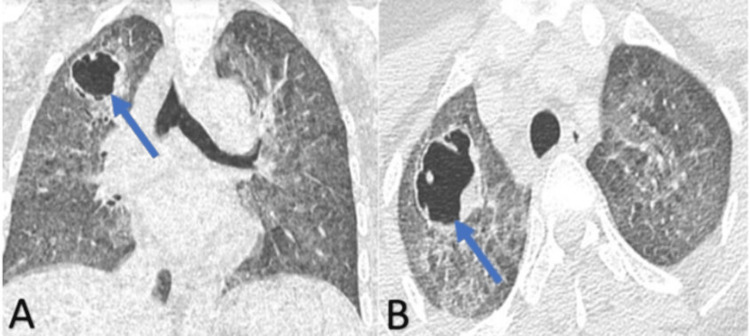
Coronal (A) and axial (B) computed tomography showing the right apical excavated lesion (arrows) with pulmonary nodules and micronodules.

A computed tomography (CT) scan of the head showed no abnormality. A CT scan of the chest revealed a right apical excavated lesion, pulmonary nodules, and mediastinal lymphadenopathy (Figure 1).

**Figure 2:**
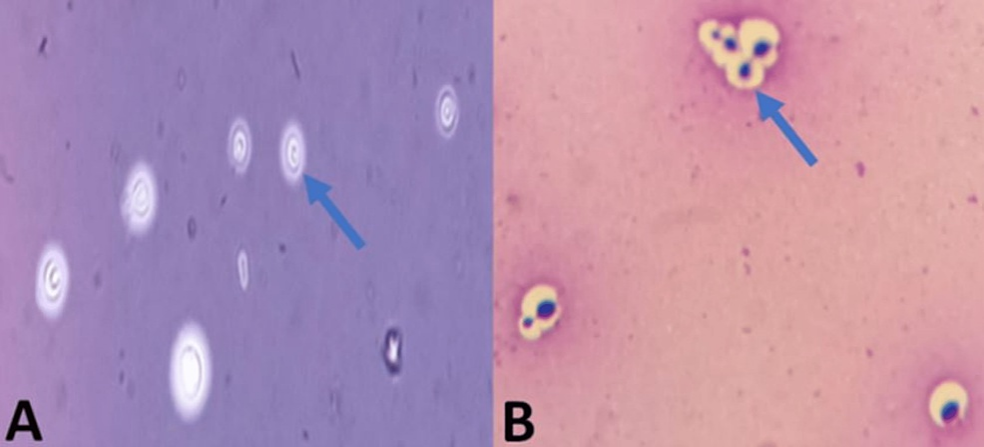
Direct microscopic examination of cerebrospinal fluid with India ink (A) and sputum stained with May-Grünwald-Giemsa stain (B) showing encapsulated yeasts (arrows).

**Figure 3:**
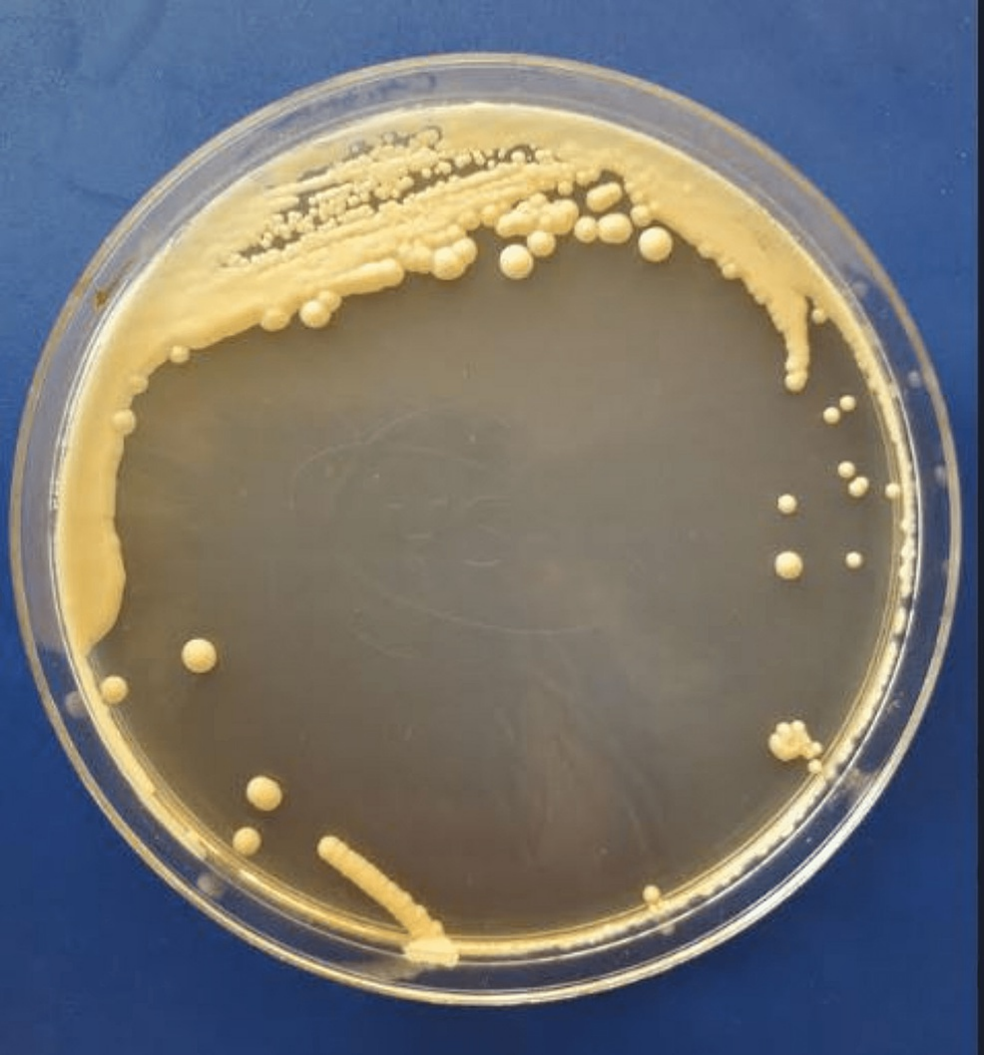
Sputum culture showing white smooth colonies of Cryptococcus neoformans in Sabouraud dextrose agar.

The patient underwent a lumbar puncture. The direct microscopic examination of cerebrospinal fluid (CSF) stained with India ink and sputum stained with May-Grünwald-Giemsa (MGG) stain showed encapsulated yeasts (Figure 2). The identification after culture on Sabouraud dextrose agar showed yeasts identified as *C. neoformans* (Figure 3). In addition, the latex agglutination test for cryptococcal polysaccharide antigen was positive in both CSF and serum. Blood culture was also positive for *C. neoformans*.

The diagnosis of disseminated cryptococcosis revealing an HIV infection was confirmed based on the neuro-meningeal and pulmonary involvement, associated with *C. neoformans* fungemia.

The patient was treated with amphotericin B and flucytosine. However, due to unresponsiveness to this treatment, fluconazole and voriconazole were started. A follow-up lumbar puncture 10 days later tested positive for India ink and cryptococcal polysaccharide antigen in CSF. Respiratory distress and fever worsened rapidly, and the patient died despite the adaptation of the antifungal treatment.

## Discussion

*Cryptococcus *is present in soil contaminated with bird excrement. Humans can get cryptococcosis by inhalation of the spores that penetrate the airways of the lung [7]. *C. gattii* infection is associated with immunocompetent individuals. *C. neoformans* infection is associated with increased mortality and is usually found in immunocompromised hosts, especially those with HIV infection, those taking immunosuppressive medications such as chronic steroids, or those who have had solid organ transplants [8].

The yeast starts by colonizing the lungs and remains latent in subpleural spaces until reactivation in case of immunosuppression. In immunocompromised patients, pulmonary cryptococcosis is generally symptomatic and can quickly lead to acute respiratory distress syndrome. After pulmonary infection, the yeast can disseminate to the blood and the CNS causing fatal meningoencephalitis. Rarely, other organs such as bones, joints, and skin may also be affected [9].

CNS involvement in immunocompromised patients is frequent. Classical meningeal manifestations are present in about 30% of the patients causing delayed diagnosis, consequently, lumbar puncture is often justified. In the case of cryptococcal meningitis in non‐HIV patients, CSF is usually clear with lymphocytic pleocytosis and elevated protein levels. In immunocompromised patients, CSF can be normal [10]. In HIV patients, India ink microscopic examination, as a quick method to reveal *Cryptococcus *in the CSF, is positive in 70-90% of cases and in 50% of non-HIV patients [11].

Cryptococcal polysaccharide antigen is detectable in serum and CSF. This test is very accurate for diagnosing invasive disease [12]. When serum is tested, the sensitivity is nearly 100%, and the specificity is between 96% and 99.5%. Concerning the CSF, sensitivity is about 100%, and specificity is between 93.5% and 99.8% [13]. Disseminated cryptococcosis is revealed by positive culture from two distinct sites or by positive blood culture [14].

The diagnosis of pulmonary cryptococcosis is made by chest imaging and by identifying the yeast in respiratory secretions, including sputum, endotracheal aspirates, or bronchoalveolar lavage. In our case, the chest CT scan showed a right apical excavated lesion, pulmonary nodules, and mediastinal lymphadenopathy. Hemoculture, sputum, and CSF culture were positive for *C. neoformans.*

The radiological signs of pulmonary cryptococcosis may mimic lung cancer or metastasis. The most common radiological findings include nodules, consolidation, pulmonary infiltrates, and lobar opacities. Other lesions can also be found in immunocompromised patients such as cavitation, pleural effusion, and lymphadenopathy [13].

Mild-to-moderate forms of pulmonary cryptococcosis can be treated with oral fluconazole at 400 mg/day for six to 12 months. Severe pulmonary, neuro-meningeal, and disseminated cryptococcosis should be managed with induction antifungal therapy, including amphotericin B at 3 mg/kg/day and flucytosine at 100 mg/kg/day for two weeks, followed by fluconazole for eight to 12 months [13,15].

Cryptococcosis is associated with poor prognosis and high mortality, especially in HIV patients. Despite antifungal therapy, about 10% to 25% of HIV patients die with cryptococcosis. Within the first year of onset of cryptococcal disease, 30% to 60% of HIV patients die [16].

## Conclusions

Cryptococcosis as an invasive and opportunistic fungal infection must be suspected in HIV patients with persistent fever, ineffective antibiotic therapy, and non-specific neurologic manifestations to support early diagnosis and adequate management.

Diagnosis of disseminated cryptococcosis with neuro-meningeal and pulmonary involvement is made by imaging studies, fungal cultures from biological fluids (blood, sputum, bronchoalveolar lavage, CSF), and cryptococcal polysaccharide antigen test.

Despite improvements in the treatment of HIV and cryptococcosis, disseminated forms remain a major cause of mortality in HIV patients.
